# The Autism Related Protein Contactin-Associated Protein-Like 2 (CNTNAP2) Stabilizes New Spines: An *In Vivo* Mouse Study

**DOI:** 10.1371/journal.pone.0125633

**Published:** 2015-05-07

**Authors:** Amos Gdalyahu, Maria Lazaro, Olga Penagarikano, Peyman Golshani, Joshua T. Trachtenberg, Daniel H. Gescwind

**Affiliations:** 1 Department of Neurobiology, Integrative Center for Learning and Memory, Semel Institute for Neuroscience and Behavior, Brain Research Institute, David Geffen School of Medicine, University of California Los Angeles, Los Angeles, CA, United States of America; 2 Department of Neurology, Semel Institute for Neuroscience and Behavior, Program in Neurogenetics and Neurobehavioral Genetics, David Geffen School of Medicine, University of California Los Angeles, Los Angeles, CA, United States of America; University of Nebraska Medical Center, UNITED STATES

## Abstract

The establishment and maintenance of neuronal circuits depends on tight regulation of synaptic contacts. We hypothesized that CNTNAP2, a protein associated with autism, would play a key role in this process. Indeed, we found that new dendritic spines in mice lacking CNTNAP2 were formed at normal rates, but failed to stabilize. Notably, rates of spine elimination were unaltered, suggesting a specific role for CNTNAP2 in stabilizing new synaptic circuitry.

## Introduction

In cortical circuits, synaptic connectivity is highly regulated and specific as only a minority of the physically possible connections between a dendrite and adjacent axons actually exist [1, 2]. This makes the gain and/or the loss of synapses (‘rewiring’) significant for neuronal circuits function [1]. Indeed, the gain, maintenance, and loss of synapses mediate learning, memory, and extinction [3–7].

Because rewiring allows adaptive behaviors, impaired rewiring may result in a variety of psychiatric disorders. Specifically, an emerging body of literature suggests that abnormal ‘rewiring’ or synaptic function is one of the main pathologies of autism spectrum disorders (ASD) [8–10]. We hypothesized that CNTNAP2, a protein whose absence is associated with ASD [11, 12], would mediate synaptic connectivity.

CNTNAP2 belongs to the NEUREXIN family that mediates synaptic cell-adhesion [13], it is present in the synaptosomal fraction [14] and knock-down of *Cntnap2* in a cortical culture impairs development of spines, the anatomical sites of most excitatory synapses [15]. However, it is unknown if CNTNAP2 mediates synaptic connectivity *in vivo*. Moreover, distinct proteins mediate synapse gain, loss, and maintenance [16–21], so defining which of these processes are influenced by CNTNAP2 is necessary for understanding CNTNAP2 molecular contribution to behavior.

## Materials and Methods

### Mice

*Cntnap2* mutant and WT mice (both males and females, age 2–5 months) were obtained from heterozygous crossings as described [12]. Mice were kept in 12 hr light/12 hr dark cycle and had ad-lib access to food and water. All procedures involving animals were performed in accordance with the UCLA animal research committee and approved by UCLA institutional animal care and use committee (IACUC), known locally as the Chancellor’s Animal Research Committee (ARC).

### Cranial window

The procedure was done as in [22], Carprofen (Pfizer 15 ug/25 g mouse) analgesia was administered subcutaneously prior to surgery and then daily for the next 4 days. Mice were anesthetized with Isoflurane (5% for induction, 1–2% thereafter), the scalp and connective tissue were removed, and the skull was covered with VetBond. An aluminum metal bar with 2 traded holes was attached to the skull with black Dental Acrylic. A 3 mm diameter craniotomy was done above part of the primary somatosensory cortex (S1) known as the barrel cortex (from Bregma: rostral −1.5, lateral 3 mm). A custom-made 3mm coverglass (Bellco Glass) was placed and sealed with VetBond cyanoacrylate glue. The dry glue was covered with Dental Acrylic. One ml Ringer solution was given subcutaneous after the surgery. During the surgery, and until full recovery, the mouse temperature was kept at 37°C using a heated plate and a rectal temperature sensor.

### Imaging

Mice with cranial window over the barrel cortex [23] were anesthetized with isoflurane (5% for induction, 1.5% thereafter) in pure oxygen. The mice were mounted in a custom-made stage using a pre-attached head bar, and their temperature was kept on 37°C using a heated plate and a rectal temperature sensor. We imaged in layers 1–3 (depth <300um) GFP-labeled neurons whose cell bodies were at layer 5b (layer 5b neurons). Neurons were imaged *in vivo* using a custom-built 2-photon laser scanning microscope using ScanImage acquisition software written in MatLab. GFP was excited at 915nm. Emitted photons were filtered with a Semrock FF01-514/30 bandpass filter and a Semrock FF01-750/SP laser blocking emission filter. Filtered photons were detected with a Hamamatsu R3896 photomultiplier tube.

### Statistical analysis

Analysis of spines was performed using ScanImage software following the guidelines established in reference [23]. The percentage of gained or eliminated spines was calculated as the number of spines added or lost between two time points, respectively, divided by the total number of preexisting spines. Significance was determined by a student t-Test. Spine dynamics and density data is presented as mean ± s.e.m.

## Results

**Fig 1:**
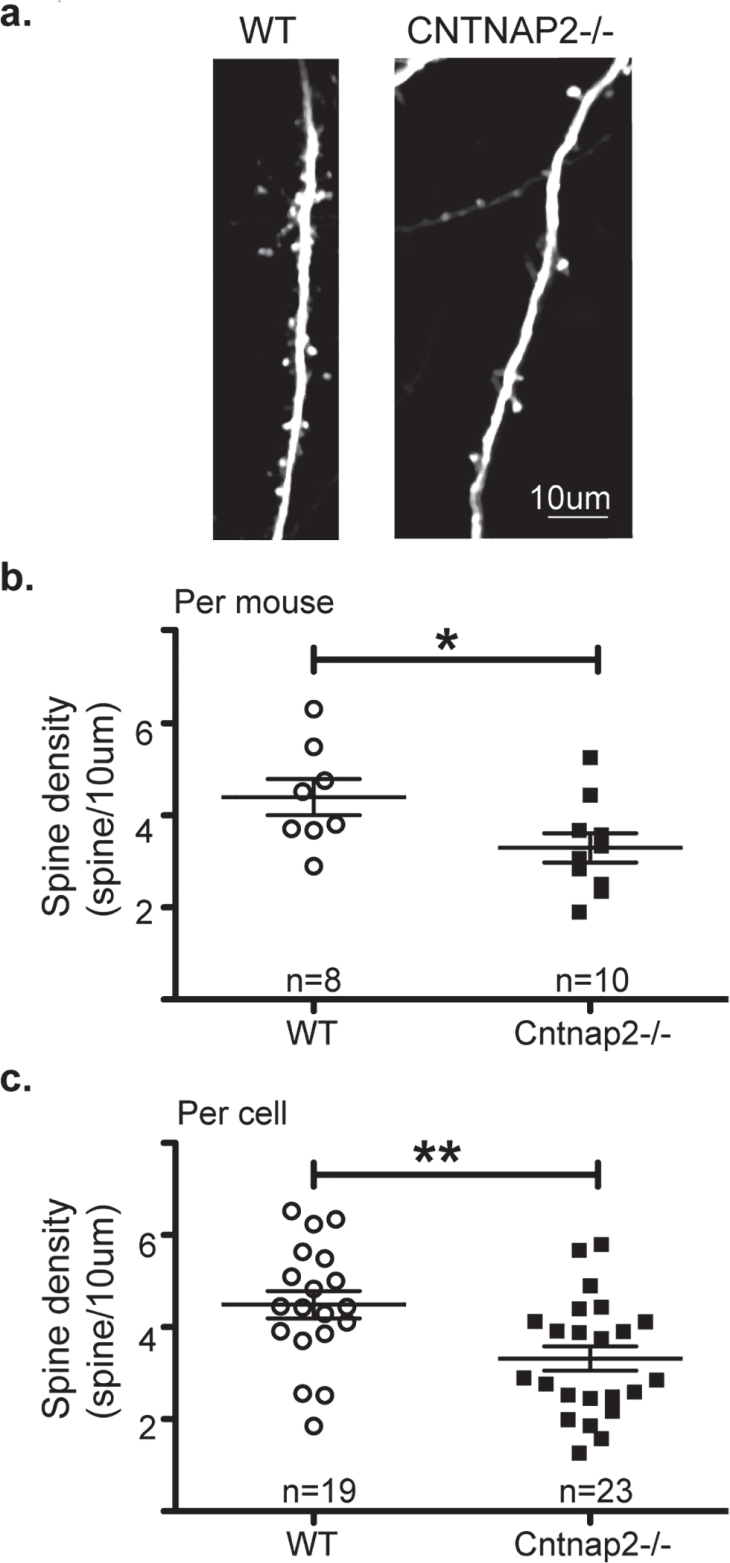
Loss of Cntnap2-/- decreases spine density. **a.** Low magnification images of dendrites and spines in WT mouse (**left)**, and in Cntnap2-/- mouse **(right)**. **b.** Quantification of spine-density. **Top plot** analysis per mouse (n = 10 Cntnap2-/- mice, n = 8 WT mice). **Bottom plot** analysis per cell (n = 23 Cntnap2-/- neurons, n = 18 WT neurons). Note the significant decrease in spine density in Cntnap2-/- mice (right) relative to WT (left).(Error bars indicate standard error (SEM), * P<0.05; **P<0.01; t-Test).

To test our hypothesis that CNTNAP2 is necessary for neuronal connectivity, we first compared the density of dendritic spines of layer 5b neurons [24] in Thy1-GFP/*Cntnap2*-/- (2582 spines, 23 cells, 10 mice) versus Thy1-GFP/WT (2139 spines, 19 cells, 8 mice) littermate controls in S1 *in vivo*. We found about 1/3 reduced spine density in *Cntnap2*-/- relative to controls (**Fig 1A,** examples of spine densities in WT and in KO; **1b** density per mouse: KO = 3.3±0.3, WT = 4.4±0.4 spine/10um P = 0.04; per cell: KO = 3.3±0.3, WT = 4.5±0.3 P = 0.005 spines/10 micron). Therefore, CNTNAP2 was necessary for proper neuronal connectivity.

**Fig 2:**
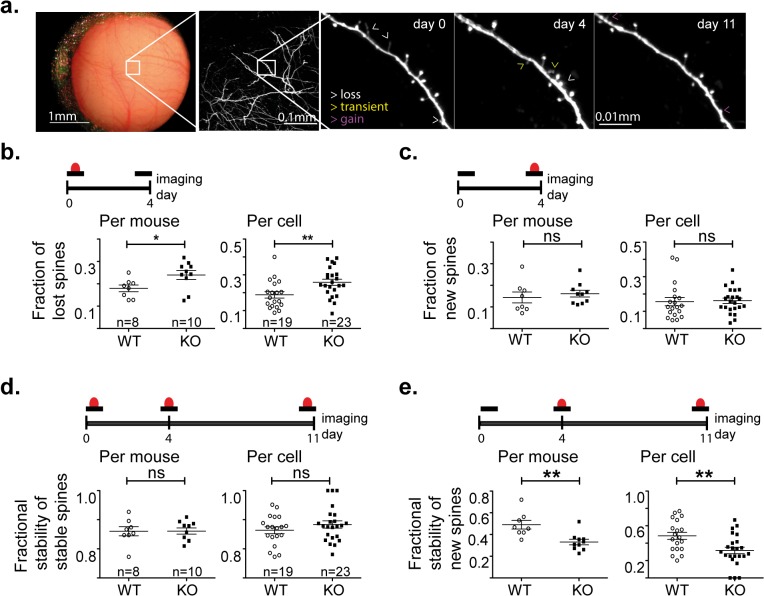
Loss of Cntnap2 decreases specifically stabilization of new spines. **a.** From left to right: Chronic imaging through a cranial window of L5 pyramidal neuron. The 3 images on the right show the dynamics of spines on a dendrite segment followed for 11 days. **b-e. Top** a spine (red) on a dendrite (black) at the indicated imaging days. **Left plots** analysis per mouse (n = 10 Cntnap2-/- mice, n = 8 WT mice). **Right plots** analysis per cell (n = 23 Cntnap2-/- neurons, n = 18 WT neurons). **b.** The fraction of spines lost during 4 days. Note the significant increase in spine loss in Cntnap2-/- mice. **c.** The fraction of spines gain. Note the absence of a significant difference between WT and Cntnap2-/- animals. **d.** The fraction of maintained spines out of the spines which were stable during the first 4 days. Note the absence of a significant difference between WT and Cntnap2-/- animals. **e.** The fraction of stable spines out of the spines gained in the first 4 days. Note the significant decrease in Cntnap2-/- mice. (Error bars indicate standard error (SEM), NS non significant; * P<0.05; **P<0.01; t-Tests).

The reduced spine density could result from a reduced spine formation or from an increase in spine elimination. To distinguish between these possibilities we imaged the same mice four days later (**Fig 2A)** and calculated the fractions of spines that were lost and gained during these four days for each mouse and for each cell. We chose a time window of four days to match former studies ([6, 25] [4]). We found a significant increase in spine loss in *Cntnap2*-/- mice versus controls, either calculated per mouse (**Fig 2B left)**, or per cell (**Fig 2B right)** (~30% difference; fractional loss per mouse: KO = 0.24±0.02, WT = 0.18±0.02, P = 0.03; per cell: KO = 0.26± 0.017, WT = 0.19±0.018, P = 0.008). In contrast, there was no significant difference in the fraction of spines gained. (**Fig 2C,** Fractional gain per mouse: KO = 0.16±0.015, WT = 0.14±0.025, P = 0.54; per cell: KO = 0.16±0.015, WT = 0.16±0.02, P = 0.85). Therefore, an increase in spine elimination in *Cntnap2*-/- mice enhanced the normal process of net-synapse-loss over time [26] and explained the reduced spine density in *Cntnap2*-/- mice versus controls.

The increased rate of spine loss can result from higher loss of formerly stable spines, or from higher loss of new spines. It is important to specify the impairment because new spines have distinct roles from stable spines [4–6]. To distinguish between these possibilities, we imaged the same 4,721 spines again at a third time point (day 11) because at that time (day 11) the survival of new spines (identified at day 4) approaches plateau [4]. Based on the first four days, we classified each spine present at day 4 either as stable (i.e. was present on day 0 and day 4) or as new (i.e. appeared at day 4) and measured its stability at day 11. We found an unchanged fraction of stable spines that remained stable (**Fig 2D**, fractional stability of stable spines per mouse: KO = 0.86±0.01, WT = 0.86±0.015, P = 0.98; per cell: KO = 0.88± 0.01, WT = 0.86±0.01, P = 0.26). In contrast, we found marked instability of new spines in *Cntnap2*-/- mice versus controls (**Fig 2E**, ~60% difference; stability per mouse: KO = 0.33±0.025, WT = 0.49±0.04, P = 0.003; per cell: KO = 0.31± 0.04, WT = 0.48±0.04, P = 0.004). Therefore, the increase of spine loss in *Cntnap2*-/- mice (Fig 2B) is caused by a specific impairment in stabilization of new spines.

## Discussion

A synapse’s life is composed of molecularly and structurally distinct stages that mediate distinct functions [16]. Dendritic filopodia search for suitable axons but most of the established connections are eliminated within hours in an activity independent mechanism. The remaining connections (~15%) become spines, most of which are lost within few days in an activity dependent process [16]. Our data suggest that CNTNAP2 is necessary for stabilization of those new spines. The surviving spines acquire RNA translation machinery, enlarge their volume, and are largely stable [16] although pruning continues especially after new experience [4–6]. Our finding that there were no changes in stable spines indicate that CNTNAP2 is not necessary for spine maintenance or pruning. Therefore, CNTNAP2 is specifically necessary for the stabilization of new synaptic contacts, a process that is thought to underlie the consolidation of adaptive behaviors [4, 7, 27]. These results are not confounded by effects of developmental plasticity because in this study we used young adult mice.

How CNTNAP2 mediates stabilization of new spines at the biochemical level is still unknown. However, CNTNAP2 interacts with the scaffold protein Calcium/calmodulin-dependent Serine protein Kinase (CASK) [28] whose knockdown reduces spine density in an hippocampal culture [29]. We speculate that CASK may function together with CNTNAP2 to promote stabilization of new spines. To our knowledge, only alphaCaMKII has been shown before to mediate long-term stabilization of specifically new spines [21], but it is unknown if there is a biochemical link between alphaCaMKII and CNTNAP2.

Our *in vivo* results are distinct from recent *in vitro* results [15], which is perhaps not surprising given the role of intact circuitry and environment including glial cells [30] in synapse development.

Abnormal synaptic connectivity has been reported in other syndromic forms of ASD such as fragile-X, and Mecp2-duplication. Together with our findings in Cntnap2, these data suggest that synaptic defects may be a common theme in many forms of ASD. Interestingly, each of these three mouse models of syndromic ASD show a distinct synaptic deficit. The rates of both spine elimination and formation are enhanced in both Fmr1-/- and in Mecp2-duplication mouse, but they balance each other in the Fmr1-/- mouse [20] and favor spine loss in the case of Mecp2-duplication [31]. In the Cntnap2-/- mouse we found an unchanged rate of spine formation but increased elimination. So, although synaptopathology may be a common result in many cases of ASD these data show very distinct synaptic defects in distinct monogenic mouse models of ASD.

Together, these data indicate that studying synaptic deficits in other genetic forms of ASD would be valuable. The further understanding of what specific processes are affected in ASD synaptopathology will help inform the development of targeted therapies.
