# Correction: The Autism Related Protein Contactin-Associated Protein-Like 2 (CNTNAP2) Stabilizes New Spines: An *In Vivo* Mouse Study

**DOI:** 10.1371/journal.pone.0129638

**Published:** 2015-05-29

**Authors:** Amos Gdalyahu, Maria Lazaro, Olga Penagarikano, Peyman Golshani, Joshua T. Trachtenberg, Daniel H. Geschwind

The sixth author’s name is spelled incorrectly. The correct name is: Daniel H. Geschwind. The correct citation is: Gdalyahu A, Lazaro M, Penagarikano O, Golshani P, Trachtenberg JT, Geschwind DH (2015) The Autism Related Protein Contactin-Associated Protein-Like 2 (CNTNAP2) Stabilizes New Spines: An *In Vivo* Mouse Study. PLoS ONE 10(5): e0125633. doi:10.1371/journal.pone.0125633
